# Exploring the Value of Technology to Stimulate Interprofessional Discussion and Education: A Needs Assessment of Emergency Medicine Professionals

**DOI:** 10.2196/jmir.3482

**Published:** 2014-06-30

**Authors:** Jennifer Riley, Melissa McGowan, Linda Rozmovits

**Affiliations:** ^1^St Michael's HospitalDepartment of Emergency MedicineToronto, ONCanada; ^2^Division of Emergency MedicineDepartment of MedicineUniversity of TorontoToronto, ONCanada; ^3^Independent Qualitative ResearcherToronto, ONCanada

**Keywords:** qualitative research, interprofessional education, technology, emergency medicine

## Abstract

**Background:**

The emergency department (ED) is an environment fraught with increasing patient volumes, competing priorities, fluctuating information, and ad hoc interprofessional clinical teams. Limited time is available to reflect on and discuss clinical experiences, policies, or research with others on the involved team. Online resources, such as webcasts and blogs, offer an accessible platform for emergency shift workers to engage in interprofessional discussion and education.

**Objective:**

Our objective was to explore the current opportunities for shared learning and discussion and to discover the potential of online resources to foster and facilitate interprofessional education within an academic tertiary emergency department community.

**Methods:**

A qualitative study using semistructured interviews was conducted to solicit participants’ views of the current culture of IPE in the ED, the potential value of introducing new online resources and technology in support of IPE, and possible barriers to uptake. Participation was voluntary and participants provided verbal informed consent.

**Results:**

Online resources discussed included webcasts, interactive discussion forums, websites, and dashboard with links to central repositories. Identified barriers to uptake of new online resources were an unwillingness to “work” off-shift, a dislike of static one-directional communication, concerns with confidentiality, and the suggestion that new resources would be used by only a select few.

**Conclusions:**

Owing to the sensitive dynamics of emergency medicine—and the preference among its professional staff to foster interprofessional discussion and education through personal engagement, in an unhurried, non-stressful environment—introducing and investing in online resources should be undertaken with caution.

## Introduction

The emergency department (ED) is an unpredictable environment characterized by competing priorities, frequent interruptions [1], and constant demands on resources, personnel, and time. The external demands from ED crowding limit educational opportunities during clinical shifts where there is little or no downtime for detailed discussion [2]. Emergency physicians, nurses, and allied health practitioners within this environment work in shifts, often of varying durations with different start times, resulting in a constantly changing landscape of staff, interprofessional clinical teams, and patients [3]. Owing to these dynamics, there is limited opportunity while working clinically in the ED to collectively discuss or debrief clinical practice and experiences. Moreover, the inherent culture of emergency medicine and the nature of shift work further limit face-to-face interactions and attendance at scheduled events. With these constraints on interprofessional education (IPE), potential gains to communication, team behaviors, and collaborative practice are lost [4,5].

One potential way to overcome the lack of time and space for shared learning in the ED is through the use of online resources. The increased connectedness that results from information and communication technologies (ICT) has overcome geographic barriers of rural or widely dispersed health practitioners, with variable access to peers, specialists, and information resources in other disciplines [6-8]. These tools can facilitate the evolution of virtual “communities of practice” (VCoP) [9], groups of individuals who share interests and expertise through online collaboration. For example, discussion forums can facilitate access to information, such as case management [10] and practice issues [11], foster communication through a collaborative and reflective environment [12-14], and serve as a repository for educational content [11] and archived discussion histories [10]. Such an online platform for knowledge management using Web 2.0 technologies was evaluated for ED physicians, nurses, and trainees and found to be a useable option for collaboration [15]. Similarly, wikis (collaboratively managed, information-based websites) have been explored as possible knowledge translation tools designed to improve patient care in trauma through access to evidence-based information and standardized protocols and leading to information sharing and teamwork [16]. However, while incorporating emerging technologies into IPE programing may address the obstacles to shared learning that exist in the ED environment, for ED staff, the introduction of any new online resources should be associated with a perceived usefulness and benefit to clinical practice [17,18]. We sought to explore the perspectives, attitudes, and receptivity of health professionals (HP) in the ED community to inform the development of online platforms for future IPE.

## Methods

### Aims

The aim of this study was to explore and describe (1) current opportunities for shared learning and discussion among emergency health care professionals, and (2) the potential value and interest in online resources to foster and improve IPE within the ED community.

### Design

This qualitative study was conducted from July to September 2012 and used semistructured interviews to solicit participants’ views of the current culture of IPE in the ED, the potential value of introducing new online resources and technology in support of IPE, and possible barriers to uptake. Participation was voluntary and participants provided verbal informed consent. Ethical approval was received from the Institutional Research Ethics Board.

### Setting

St Michael’s Hospital Emergency Department is an urban, academic, tertiary care center that serves over 72,000 patient visits and approximately 650 trauma team activations annually.

### Sampling and Recruitment

All current emergency department physicians, nurses, and allied health professionals (25 medical doctors, 77 full-time registered nurses, and 1 social worker) were invited to participate via email by the study coordinator. Purposive sampling supplemented by snowball sampling was used to capture a range of professions, years in practice, and gender.

### Qualitative Data Collection

Semistructured, one-to-one interviews were conducted by an experienced, independent qualitative researcher not associated with the emergency department. All interviews were digitally audio-recorded for verbatim transcription. Interviews took place either in person or by telephone at the convenience of the participant.

### Qualitative Analysis

All transcripts were checked against sound files for accuracy and corrected where necessary. Data were then entered into HyperResearch software for qualitative data analysis. A coding structure was developed in discussion with the research team. For the analysis, the method of constant comparison was used [19] including searches for disconfirming evidence.

## Results

### Summary

Twelve semistructured one-to-one interviews were conducted over a 6-week period between August and September 2012 with a range of years of practice and professions (6 registered nurses, 5 medical doctors, and 1 social worker) represented (Table 1). Interviews lasted between 17 and 34 minutes with an average length of 27 minutes. Three were conducted in person, and the remainder were conducted by telephone.

**Table 1:** Demographics of ED participants.

		n
**Sex**		
	Male	4
	Female	8
**Age**		
	20-29	1
	30-39	5
	40-49	5
	50-59	1
**Years in emergency medicine**		
	<1-5	3
	6-10	4
	11-15	3
	16-20	1
	>20	1
**Years at study institution**		
	<1-5	4
	6-10	3
	11-15	4
	16-20	0
	>20	1

### Current Culture of Shared Learning

Although participants reported good working relationships within and between professions, this did not necessarily translate into a culture of shared learning. Discussion was described as occurring within rather than between professional groups and tended to be informal, brief, and accessed on an ad hoc basis, leaving little if any time for substantive debriefing or unhurried reflection. For example, “I would say, for the most part...the culture is you tend to bounce things off of another physician, maybe because they’re sort of in the same position as you and have the same role, I guess in the same way like nurses tend to talk to each other” [ED 12].

In addition, time pressure and increasingly demanding workloads engendered a sense of apathy among many emergency providers and affected staff willingness to attend education and/or debriefing sessions on days when they were not working:

I think there’s workload issues…Sometimes the department is busier…doing anything else when you leave here, like, you’re just totally fried. And then coming in on your day off to, like, go and do something is also a bit of a challenge, right? So I think there’s a lot of barriers.

ED 07

### Perceived Potential Benefits of New Interprofessional Education Opportunities

Overall, participants recognized that IPE held the potential to enhance team dynamics and patient care and safety by providing the chance to learn from errors and by improving understanding of other HP roles and perspectives: “any time you share an experience with the people that you work with it only makes your cohesion, your communication, the way you work, better together” [ED 07].

In addition, some participants felt that IPE could address inequity in learning opportunities because current formal meetings and rounds were predominantly physician-driven due to scheduling and historical culture. More importantly, many participants felt that improving discussion across the ED would foster engagement, lead to higher levels of job satisfaction and mutual support, and help thwart burnout: “I just think the burnout is kind of there and it potentially could prevent some of it, and just lead to more job satisfaction which, of course, would improve patient care” [ED 06].

### Acknowledging Emergency Department Culture

Although some participants attached little value to proposed IPE opportunities—believing that the current culture was adequate to meet their needs—others saw merit in the idea but doubted they would participate in new initiatives because they were so habituated to the current culture. In order to engage staff, participants felt that any new learning opportunities should be substantially “value-added”, should address compelling subject matter, and directly affect clinical practice. Many also argued emphatically that new resources should consolidate and replace those currently in existence to encourage uptake in already overburdened ED staff. Finally, skepticism about the value and effectiveness of IPE due to inherent professional and hierarchical differences among emergency professionals must be acknowledged:

So interprofessional learning is hot and it’s going to be hot for two or three more years until we find the new interprofessional learning...I’m not closing my mind to it, I just don’t know what the value is in me learning from other people downstream or upstream of me. I don’t know what I’m supposed to do with that.

ED 05

### Enhancing Interprofessional Education With Technology

#### Overview

The potential utility of online resources and technology to address the limitations of shared learning in the ED environment was explored. Participants commented on a variety of possible online resources including webcasts, discussion forums, and a knowledge archive.

#### Webcasts

Participants’ responses to the proposed use of webcasts were mixed. Although many saw value in the flexibility enabled by webcasting (such as adding value in the workplace during downtime and eliminating the issues and cost associated with commuting and/or childcare), they also expressed doubt about ever accessing such a resource because of its impersonal and unidirectional nature and the limited access to computers in the workplace.

#### Discussion Forums

A range of views was expressed about the value of an online discussion forum as a vehicle for enabling discussion and shared learning. Although discussion forums were preferred over webcasting because of their interactive nature, asynchronous features, and potential for sharing ideas and questions about evolving clinical practice and new research, many felt such interfaces would be adopted by only a few individuals because participation during personal time was a critical factor for success, interaction, and sustainability. Significant concerns about confidentiality and the protection of patient information were also raised: “the problem is how private can you be, because you can just screenshot anything in your computer and send that around…it really becomes a bit tricky” [ED 02].

In order for a discussion forum to be genuinely beneficial in the eyes of the emergency health care professionals, it would have to be secure and well moderated with clearly defined goals for particular types of exchange.

#### Knowledge Archive

A technology-based resource that held the greatest appeal for participants was a departmental website that would serve as a centralized repository for information and documents critical to patient flow in an emergency department, such as clinic forms, policies, protocols, and important and frequently accessed contact numbers. This kind of centralized “hub” would potentially consolidate information of value to all team members and improve on the current, rather haphazard system that was not easily, consistently or universally accessible:

Taking these 58 fragments of how we’re supposed to do things, whether it’s on a piece of paper stuck to the wall, an old email, on a print-out that’s in the doctors’ office, what somebody said this week…one common base for all of it. That would be my ideal.

ED 05

Once established, it was suggested that additional, interactive components, such as a blog or discussion forum, could then evolve: “if the website had other things that we need for work…different policies or things we might have to look up, or the clinic referral forms…it’d be convenient enough that we would use it during work, then you could probably tag on, say, a blog there” [ED 12].

#### Consideration for Investment in Interprofessional Education With Technology

Engaging in work during off hours was viewed as undesirable by most participants, despite the value attached to enhancing practice and patient care through participation in technology-assisted IPE. The nature and culture of emergency medicine with long shifts and fast-paced, unpredictable clinical volumes and patient acuities reinforced the notion that time away from work was a precious commodity: “people I think work so hard here that it’s...they give so much that they just...they’re done” [ED 01].

Moreover, many participants were concerned about continued investment in tools and resources that did not fill a need and did not consolidate or replace what was currently available. In response to suggestions that investing in technology would foster new and greater IPE opportunities, many felt that face-to-face interaction was preferable. This was partly owing to the sensitive nature of some discussions and partly because they valued the opportunity to interact directly with colleagues in an unhurried, non-stressful manner:

We’re human beings…no matter the way the world is changing, we communicate face-to-face and it’s important to be able to read facial expressions, body language when we’re sharing things. I just think having the warm body in the room makes the experience that much more valuable. I can type my feelings out in a discussion board but I’m certain that it wouldn’t have as much value for me.

ED 03

## Discussion

### Principal Findings

The findings of this study revealed a culture of collegial working relationships between professions in the ED and the recognition that interprofessional collaboration fosters communication, positively impacts team dynamics, and in turn improves patient care and safety. However, similar to the findings of Creswick et al [20], shared learning occurrences in the ED were found to be “siloed” within professions. This was evident both in the clinical arena and in educational rounds, which are more readily offered and accessible to physicians. New IPE ventures must address these current inequities and build upon the natural rapport between professions in the clinical emergency setting [3] to benefit all.

Important considerations in the development and implementation of technology-based IPE opportunities for emergency HPs were identified. To facilitate maximal uptake, new resources must consolidate and unambiguously replace previously existing resources and, more importantly, make processes easier and more efficient rather than becoming an additional burden. Creating new online resources that overlap with current, partially abandoned resources is more likely to engender skepticism than engagement among emergency HPs and to exacerbate “change fatigue”. Demonstrable enhancement of practice in the ED before implementation must be considered. As Ayatollahi et al [17] showed, the perceived usefulness of technology was a more powerful predictor of positive attitude than actual ease of use. Likewise, usability and trust in information and communication technology have been shown to be critical factors for the success of virtual communities of practice [21].

The most well-received proposal to enhance shared learning and discussion through the use of technology was for a departmental website, initially serving as a knowledge archive for frequently needed clinical resources. Online repositories are not novel and have been successfully deployed as platforms for knowledge management [22], including among emergency medicine staff and trainees as an alternative portal to traditional lectures and rounds [15]. Given the potential of the website to add value to the emergency HP shift experience and flow, successful implementation and uptake may enable the addition of future, interactive components beyond its initial purpose as demonstrated by Reid et al [23]. They described an intervention initially established to enhance communication with an online discussion board that evolved into a repository of documents, presentations, and images, in addition to a value-added tool to improve operations with group emails, scheduling, and portfolios.

The findings of our study suggest that introduction of technology to create more space for discussion and shared learning among emergency HPs should be undertaken with caution. The importance of face-to-face interaction among emergency staff cannot be discounted because many topics are sensitive in nature, and non-verbal cues are important to the dynamics of interaction, discussion, and ultimately shared learning [21]. Platforms such as webcasts and discussion forums, which also create potential space for shared learning and discussion were largely considered impersonal and unlikely to be used by the collective. This caution should be heeded as Tse and Wise [24] found that while most participants in an online discussion forum logged into the resource, two-thirds did not post any comments because of self-consciousness about posts and a lack of time due to competing demands—considerations translatable to the ED.

A critical factor for the success of virtual communities of practice (VCoP) is highly relevant or “value-added” content. However, this alone may still not be enough to encourage uptake of online resources. Dube found that despite including highly relevant topics, VCoP have failed because members did not see value in moving from the informal network to one that is online [18], another consideration when planning online IPE.

Moreover, in an era of heightened privacy and increasing legislative protection of information, many emergency HPs were concerned about the challenge of maintaining confidentiality [25]. The process for discussing case management or practice in an online setting, where anything posted on the Web can be reproduced and circulated without restriction with a simple screenshot, is a serious matter that must be addressed in advance of any implementation. Foong and McGrouther [11] discuss this and suggest limiting access to a selected group of users, but recognize that this is insufficient protection. Reid et al [23] also emphasize that information posted online must be considered to be in the public domain, and patient confidentiality must be preserved. These are important aspects to consider in relation to an initial knowledge archive to share and learn from experiential information and to any subsequent interactive discussion forum.

Finally, difficulties with sustaining the technologies were raised, including updating and moderating the resource. Curran-Smith and Best [12] identified skilled facilitators as pivotal to sustaining and promoting meaningful discussion with Web-based continuing education, while Archambault et al [16], when developing an online wiki for trauma, were faced with the frequency of changing information and the potential difficulties with keeping the site updated. These considerations have clear resource implications, which may pose further challenges to implementation.

### Limitations

Our study was conducted within a single emergency department. Variations in culture between institutions, departments, and HPs could yield differing perceptions of current opportunities for technology-enhanced IPE and perceived barriers to participation. As this was a small-scale exploratory study intended primarily to inform proposals for possible technology-based IPE initiatives in the ED, indications of data saturation were beginning to emerge but were not fully consolidated after 12 interviews.

### Conclusions

Introducing online resources in the ED to support IPE and discussion should be viewed with caution. New opportunities must fill a clearly defined need, be value-added, and enhance clinical practice through consolidating and simplifying existing resources. Creating a collaborative website to improve process and function may lead to a future interactive resource for shared learning across professions.

## Abbreviations

ED: emergency department
HP: health professionals
ICT: information and communication technologies
IPE: interprofessional education
VCoP: virtual communities of practice
